# The impact of chemotherapeutic drugs on the CYP1A1-catalysed metabolism of the environmental carcinogen benzo[*a*]pyrene: Effects in human colorectal HCT116 *TP53(+/+)*, *TP53(+/−)* and *TP53(−/−)* cells

**DOI:** 10.1016/j.tox.2018.02.006

**Published:** 2018-04-01

**Authors:** Alexandra J. Willis, Radek Indra, Laura E. Wohak, Osman Sozeri, Kerstin Feser, Iveta Mrizova, David H. Phillips, Marie Stiborova, Volker M. Arlt

**Affiliations:** a Department of Analytical, Environmental and Forensic Sciences, MRC-PHE Centre for Environment and Health, King’s College London, 150 Stamford Street, London SE1 9NH, United Kingdom; b Department of Biochemistry, Faculty of Science, Charles University, Albertov 2030, 128 40 Prague 2, Czech Republic; c NIHR Health Protection Research Unit in Health Impact of Environmental Hazards at King's College London in partnership with Public Health England, London, 150 Stamford Street, London SE1 9NH, United Kingdom

**Keywords:** BaP, benzo[*a*]pyrene, BPDE, benzo[*a*]pyrene-7,8-dihydrodiol-9,10-epoxide, CYP, cytochrome P450, DMSO, dimethyl sulfoxide, HPLC, high performance liquid chromatography, mEH, microsomal epoxide hydrolase, PAH, polycyclic aromatic hydrocarbon, ROS, reactive oxygen species, Benzo[*a*]pyrene, Tumour suppressor p53, Cytochrome P450, Cisplatin, Etoposide, Ellipticine

## Abstract

Polycyclic aromatic hydrocarbons such as benzo[*a*]pyrene (BaP) can induce cytochrome P450 1A1 (CYP1A1) via a p53-dependent mechanism. The effect of different p53-activating chemotherapeutic drugs on CYP1A1 expression, and the resultant effect on BaP metabolism, was investigated in a panel of isogenic human colorectal HCT116 cells with differing *TP53* status. Cells that were *TP53(+/+*), *TP53(+/–)* or *TP53(*–/–*)* were treated for up to 48 h with 60 μM cisplatin, 50 μM etoposide or 5 μM ellipticine, each of which caused high p53 induction at moderate cytotoxicity (60–80% cell viability). We found that etoposide and ellipticine induced CYP1A1 in *TP53(+/+*) cells but not in *TP53(*–/–*)* cells, demonstrating that the mechanism of CYP1A1 induction is p53-dependent; cisplatin had no such effect. Co-incubation experiments with the drugs and 2.5 μM BaP showed that: (*i*) etoposide increased CYP1A1 expression in *TP53(+/+*) cells, and to a lesser extent in *TP53(–*/–*)* cells, compared to cells treated with BaP alone; (*ii*) ellipticine decreased CYP1A1 expression in *TP53(+/+*) cells in BaP co-incubations; and (*iii*) cisplatin did not affect BaP-mediated CYP1A1 expression. Further, whereas cisplatin and etoposide had virtually no influence on CYP1A1-catalysed BaP metabolism, ellipticine treatment strongly inhibited BaP bioactivation. Our results indicate that the underlying mechanisms whereby etoposide and ellipticine regulate CYP1A1 expression must be different and may not be linked to p53 activation alone. These results could be relevant for smokers, who are exposed to increased levels of BaP, when prescribing chemotherapeutic drugs. Beside gene-environment interactions, more considerations should be given to potential drug-environment interactions during chemotherapy.

## 1 Introduction

**Fig. 1:**
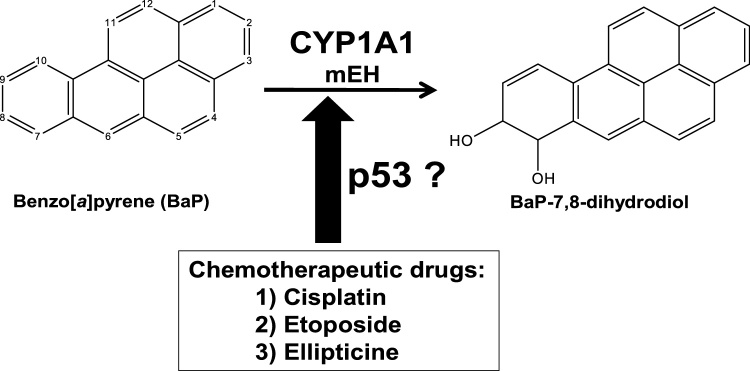
Oxidation of BaP by CYP1A1 to BaP-7,8-dihydrodiol and the possible influence of chemotherapeutic drugs – cisplatin, etoposide and ellipticine – on CYP1A1 expression in a p53-dependent manner. See text for details.

The polycyclic aromatic hydrocarbon (PAH) benzo[*a*]pyrene (BaP) is a ubiquitous environmental pollutant produced from the incomplete combustion of organic material and has been classified by the International Agency for Research on Cancer as a human carcinogen (Group 1) (IARC, 2010). Except for smokers the predominant route of human exposure to BaP is via the diet, but BaP exposure due to ambient air pollution is also of great concern (Phillips, 1999; Phillips and Venitt, 2012). BaP needs to be metabolically activated in order to exert its carcinogenic effects (Labib et al., 2016; Long et al., 2016; Long et al., 2017; Zuo et al., 2014). The metabolism of BaP is mainly catalysed by cytochrome P450 (CYP) enzymes (Reed et al., 2018), predominantly CYP1A1 and CYP1B1 (Luch and Baird, 2015). This first leads to the formation of BaP-7,8-epoxide, which is quickly metabolised by microsomal epoxide hydrolase (mEH) to BaP-7,8-dihydrodiol (Fig. 1) (Stiborova et al., 2016; Sulc et al., 2016). BaP-7,8-dihydrodiol can be further activated by CYP1A1 generating BaP-7,8-dihydrodiol-9,10-epoxide (BPDE) which is capable of reacting with DNA (Arlt et al., 2015; Kucab et al., 2015; Stiborova et al., 2016). The DNA adduct formed by BPDE is predominantly formed at the N^2^ position of guanine [i.e. 10-(deoxyguanosin-*N*^2^-yl)-7,8,9-trihydroxy-7,8,9,10-tetrahydro-BaP (dG-*N*^2^-BPDE)] (Arlt et al., 2008) and preferentially leads to the induction of G to T transversion mutations (Alexandrov et al., 2016; Kucab et al., 2015; Nik-Zainal et al., 2015). Alternatively, BaP-7,8-dihydrodiol can be activated by aldo-keto reductases leading to BaP-7,8-dione which is also capable of forming DNA adducts and generating oxidative damage to DNA (Penning, 2014).

The tumour suppressor *TP53*, which encodes the protein p53, is often called the ‘guardian of the genome’ due to its protective role in response to DNA damage and cellular stress (Goldstein et al., 2011). It is inactivated by mutation in more than 50% of human tumours, highlighting the importance of its role in normal cellular functions (Kucab et al., 2010). p53 is known most for its role in cell cycle arrest, DNA repair and apoptosis but new functions for p53 are still being discovered. Studies in our group have demonstrated a role for p53 in influencing xenobiotic metabolism (Hockley et al., 2008; Simoes et al., 2008; Krais et al., 2016a; Krais et al., 2016b; Wohak et al., 2016). Specifically, we found that BaP-induced CYP1A1 expression depends on p53 function. Using a panel of isogenic colorectal HCT116 cells with differing *TP53* status we found that BaP-induced DNA adduct formation (dG-*N*^2^-BPDE) was substantially higher in HCT116 *TP53(+/+*) cells than in *TP53(+/*–*)* and *TP53(–/–)* cells (Wohak et al., 2016). Higher DNA adduct levels in *TP53(+/+*) cells correlated with higher levels of BaP metabolites (e.g. BaP-7,8-dihydrodiol) and higher CYP1A1 protein expression relative to BaP-treated *TP53(*–/–*)* cells. Further, our findings indicated that CYP1A1 expression can be regulated through p53 binding to p53 response elements in the *CYP1A1* regulatory region, leading to increased transcriptional induction of *CYP1A1* (Wohak et al., 2016).

Most anti-cancer treatment regimens are composed of several drugs with at least one being a p53-activating drug (Goldstein et al., 2013). As treatment with chemotherapeutic drugs can also stimulate p53 expression in normal cells, based on our recent finding showing the impact of p53 function on the CYP1A1-mediated bioactivation of BaP, drug-environment interactions also need to be carefully considered. Since human exposure to BaP is almost impossible to avoid, any relationship found between chemotherapeutic drugs and BaP activation could have important health implications for patients receiving treatment for cancer, particularly for tobacco smokers.

In this study three chemotherapeutic drugs have been used: cisplatin, etoposide and ellipticine. They are all commonly used chemotherapeutic drugs that treat a variety of cancers and all have different mechanisms of cytotoxicity. Cisplatin is a platinum-containing drug used to treat testicular, ovarian, bone, and head and neck cancers, primarily by causing intrastrand crosslink DNA adducts and subsequently apoptosis (Florea and Busselberg, 2011; Siddik, 2003). The platinum atom in cisplatin reacts with nucleophilic N7 sites in adenine and guanine to form intrastrand crosslinks between the bases, with 1,2-GG-intrastrand crosslinks being the most common. Cisplatin-induced DNA damage also activates p53, which in turn promotes reactive oxygen species (ROS)-dependent p38alpha MAPK pathway activation, which causes apoptosis (Bragado et al., 2007). Etoposide is administered to treat lymphoma, lung, ovarian and testicular cancers by interaction with topoisomerase II (Montecucco and Biamonti, 2007). It is a topoisomerase poison causing single or double strand breaks, eventually promoting p53-mediated apoptosis (Karpinich et al., 2002). Besides CYP3A4/5-catalysed reactions, etoposide can be metabolised to O-demethylated metabolites by prostaglandin synthase or myeloperoxidase; these metabolites (catechol and quinone) are also topoisomerase II poisons (Yang et al., 2009). Ellipticine is used to treat osteolytic breast cancer metastases, kidney cancer, brain tumours and acute myeloblastic leukaemia (Stiborova and Frei, 2014). It elicits its anti-cancer effects predominantly through intercalation into DNA and inhibiting topoisomerase II (Stiborova et al., 2006), similar to the mechanism of action of etoposide. Ellipticine also forms DNA adducts after metabolic activation (Stiborova et al., 2014a). The main enzymes responsible for the bioactivation of ellipticine are CYP1A1, CYP1A2 and CYP3A4 (Frei et al., 2002; Stiborova et al., 2004), converting it into 12-hydroxy- and 13-hydroxyellipticine, which can then covalently bind to DNA forming adducts (Stiborova et al., 2014a). Ellipticine is also metabolised by the same CYP enzymes to form 7-hydroxy- and 9-hydroxyellipticine which are considered to be detoxication metabolites (Stiborova et al., 2014a).

The aim of the present study was to investigate whether the p53-activating chemotherapeutic drugs cisplatin, etoposide and ellipticine can influence CYP1A1 expression and whether they could potentially influence the CYP1A1-mediated metabolism of BaP. These experiments were carried out in three isogenic human colorectal HCT116 cell lines that differ only with respect to their *TP53* status: wild-type for p53 (hereafter termed *TP53(+/+*) cells), heterozygous for p53 (termed *TP53(+/*–*)* cells), and a complete knock-out of p53 (termed *TP53(*–/–*)* cells). Cells were treated with cisplatin, etoposide or ellipticine alone or in combination with BaP. Expression of DNA damage response proteins (e.g. p53 and p21) and expression of CYP1A1 and CYP3A4 was determined by Western blotting. BaP bioactivation (formation of BaP-7,8-dihydrodiol) was evaluated by high performance liquid chromatography (HPLC).

## 2 Materials and methods

### 2.1 Carcinogens and drugs

Benzo[*a*]pyrene (BaP; CAS no. 50-32-8; purity ≥96%), cisplatin (CAS no. 15663-27-1, crystalline) and ellipticine (CAS no. 519-23-3; purity ≥98%) were obtained from Sigma-Aldrich. Etoposide (CAS no. 33419-42-0; purity ≥98%) was obtained from Cayman Chemical. The BaP metabolite (±)-*trans*-7,8-dihydroxy-7,8-dihydro-BaP (BaP-7,8-dihydrodiol) that was used as a standard for HPLC was synthesised at the Biochemical Institute for Environmental Carcinogens using earlier published methods (Platt and Oesch, 1983; Yagi et al., 1977). Mass spectrometry data and high field ^1^H NMR spectra (400 MHz) for BaP-7,8-dihydrodiol were in essential agreement to those published previously.

### 2.2 Cell culture and treatment

Cells expressing either wild-type p53 [HCT116 *TP53(+/+*)], heterozygous p53 [HCT116 *TP53(+/*−)] or with a complete knockout of p53 [HCT116 *TP53(−/*−)] (Sur et al., 2009) were kindly provided by Prof. Bert Vogelstein, Johns Hopkins University School of Medicine, Baltimore, MD. HCT116 cells were grown in complete growth medium: Dulbecco’s modified Eagle’s medium (Invitrogen) with 10% foetal bovine serum (Invitrogen), supplemented with 100 units/mL penicillin and 100 μg/mL streptomycin, as adherent monolayers (Wohak et al., 2016). Cells were cultured at 37 °C in 5% CO_2_.

*TP53(+/*−) and *TP53(−/*−) cells were seeded at 3 × 10^4^ cells/cm^2^ and *TP53(+/+*) cells were seeded at 2.8 × 10^4^ cells/cm^2^ and grown for 48 h prior to treatment. Cells were then treated with the test compounds or solvent vehicle as control for up to 48 h. Etoposide and ellipticine were dissolved in dimethyl sulfoxide (DMSO), whereas cisplatin was dissolved in a 0.9% NaCl solution. The DMSO concentration was always kept at ≤0.5% of the total culture medium volume and the NaCl concentration was equal to the highest concentration in the test compound used. The final incubation volume was 150 μL medium per well (96-well plates) or 5 mL medium per 25 cm^2^ flask.

Based on previous experiments (Hockley et al., 2008; Wohak et al., 2016), cells were treated with 2.5 μM BaP to study the effects of chemotherapeutic drug-induced CYP1A1 expression and BaP metabolism in co-incubation experiments. BaP was dissolved in DMSO and kept at ≤0.5% of the total culture medium volume. For BaP co-incubation experiments, concentrations of 60 μM cisplatin, 50 μM etoposide and 5 μM ellipticine were selected (see 2.3). Cells were seeded in 25 cm^2^ flasks as described above and after 48 h cells were pre-treated with the drug for 6 or 24 h, followed by co-treatment with the drug plus 2.5 μM BaP for another 24 h.

### 2.3 Determination of cytotoxicity using crystal violet staining

The cytotoxicity of etoposide, cisplatin and ellipticine was determined in all three HCT116 cell lines in order to establish concentrations that resulted in 60–80% cell viability after 48 h. These experiments were conducted in 96-well plates at least in triplicate, and usually 8 wells were tested per condition in one assay. Concentrations of 0, 10, 25, 35, 50, 60, 75 and 100 μM cisplatin, 0, 10, 50 and 100 μM etoposide and 0, 1, 5 and 10 μM ellipticine were tested and cell viability was determined using the crystal violent staining assay (Dooley et al., 1994; Kucab et al., 2012). Crystal violet (4-[(4-dimethylaminophenyl)-phenyl-methyl]-*N*,*N*-dimethyl-aniline; Sigma) is a dye that stains DNA. The relative density of an adherent cell culture is a function of the amount of crystal violet staining, measured as absorbance at 595 nm. After 24 or 48 h treatment, the medium was removed, the cells were washed with phosphate-buffered saline (PBS) and subsequently 0.1% crystal violet in 10% ethanol was added to the wells. After a 10-min incubation the cells were washed with PBS and left to dry. Once dry the stained cells were dissolved in 50% ethanol and the absorbance of crystal violet was measured at 595 nm on a BioTek ELx800 microplate reader. Cell viability was expressed as a percentage of the control. Each assay was repeated in at least 3 independent experiments.

### 2.4 Western blotting to measure protein expression

For Western blot analysis cells were seeded in 25 cm^2^ flasks. After treatment the cells were washed with PBS twice and then lysed with 600 μL of lysis buffer (62.5 mM Tris [pH 6.8], 1 mM EDTA [pH 8.0], 2% sodium dodecyl sulphate [SDS], 10% glycerol). Cells were sonicated and centrifuged for 5 min at 10,000 rpm. Then the protein concentration of the supernatant was determined using the bicinchoninic acid (BCA) protein assay (Pierce, Thermo Scientific) according to the manufacturer’s instructions. β-Mercaptoethanol (Sigma) was added to the lysates to reduce disulphide bonds as previously described (Wohak et al., 2016). Lysates were then denatured at 90 °C for 5 min and equal amounts of protein (10 μg when probing for p53 and p21 and 20 μg when probing for CYP1A1) were separated by SDS-polyacrylamide gel electrophoresis (SDS-PAGE) using 4–12% Bis-Tris gradient gels, and Western blotted as reported previously (Kucab et al., 2012).

The membrane was blocked in 3% nonfat milk (dissolved in Tris-buffered saline [TBS] with 0.2% Tween-20) for at least 1 h at room temperature, and then incubated overnight or over 2 nights, depending on the strength of the antibody, at 4 °C with primary antibodies or anti-serum diluted in blocking solution containing 0.1% sodiumazide. The following primary antibodies and dilutions were used: anti-p53 1:2000 (Ab-6, Calbiochem) and anti-p21 (CDKN1A) 1:2000 (556431, BD Pharmingen). Anti-CYP1A1 raised in rabbits against purified human recombinant CYP1A1 was a generous gift from Prof. F. Peter Guengerich (Vanderbilt University, USA) and was diluted 1:4000 (Wohak et al., 2016). Anti-CYP3A4 1:1000 (sc-53850) was from Santa Cruz Biotechnology. The antibodies to detect β-Actin 1:20,000 (ab6276, Abcam) or GAPDH 1:20,000 (MAB374, Chemicon) were used as loading controls. The secondary horseradish peroxidase-linked antibodies were as follows: anti-mouse (170–5047; 1:10,000) and anti-rabbit (170–5046; 1:10,000) from BioRad. The membranes were then treated with SuperSignal™ West Pico Chemiluminescent Substrate (Thermo Scientific) and developed using Amersham Hyperfilm ECL (GE Healthcare) to detect protein expression.

The antibody CYP1B11-A (Alpha Diagnostic International,) previously shown to detect human CYP1B1 in BaP-treated MCF-7 human breast carcinoma cells (Hamouchene et al., 2011) was tested but did not detect CYP1B1 in HCT116 whole cell lysates (data not shown).

### 2.5 HPLC analysis of BaP, ellipticine and etoposide metabolites

For the analysis of BaP, ellipticine and etoposide metabolites, culture medium from exposed cells was collected centrifuged for 5 min at 300*g* at 4 °C and stored at −80 °C until needed for further processing. Per sample, 1 mL of medium was extracted twice with 1 mL of ethyl acetate and 5 μL of 1 mM phenacetin was added as an internal standard. For the analysis of BaP metabolites, extracts were evaporated to dryness and dissolved in 30 μL of 100% methanol, of which 20 μL aliquots were injected on HPLC. HPLC analysis was performed using a HPLC Agilent 1100 System (Agilent Technologies) with a SunFire™ C18 reverse phase column (250 × 4.6 mm, 5 μm; Waters). The conditions used for the chromatographic separation of BaP metabolites were as follows: mobile phase A: 50% acetonitrile in water (*v*/*v*), mobile phase B: 85% acetonitrile in water (*v*/*v*). The separation started with an isocratic elution of 1.4% of mobile phase B. Then a linear gradient to 98.5% of mobile phase B in 34.5 min was followed by isocratic elution for 6 min, a linear gradient from 98.5% to 1.4% of mobile phase B in 3 min, followed by an isocratic elution for 1.5 min. Total run time was 45 min at a flow rate of 1 mL/min. The metabolites were analysed by fluorescence detection (0–6 min excitation 341 nm, emission 381 nm and 6–45 min excitation 380 nm, emission 431 nm).

For the analysis of ellipticine metabolites, extracts were evaporated to dryness and dissolved in 25 μL of 100% methanol, of which 20 μL aliquots were injected on HPLC. The column used was a 5-μm Ultrasphere ODS (4.6 × 250 mm; Beckman, Fullerton, CA), the eluent was 64% methanol plus 36% of 5 mM heptane sulfonic acid containing 32 mM acetic acid in water with a flow rate of 0.7 mL/min, and UV detection was at 296 nm. The metabolite peak areas were calculated relative to the peak area of the internal standard (phenacetin).

For the analysis of etoposide metabolites, extracts were also evaporated to dryness and dissolved in 25 μL of 100% methanol, of which 20 μL aliquots were injected on HPLC. HPLC analysis was performed on a Nucleosil^®^ C18 reversed phase column, (250 × 4 mm, 5 μm; Macherey Nagel, Germany) using a Dionex system consisting of a pump P580, a UV/Vis detector UVD 170S/340S, an ASI-100 automated sample injector, a thermobox column oven LCO 101 and an in-line mobile phase degasser Degasys DG-1210 Dionex controlled with Chromeleon™ 6.11 build 490 software. HPLC conditions were 50% acetonitrile in HPLC water (*v*/*v*), with a linear gradient from 50% to 57% acetonitrile in 7 min, and then a linear gradient from 57% acetonitrile to 50% acetonitrile in 1 min, followed by an isocratic elution of 50% acetonitrile for 1 min. Detection was by UV absorbance at 254 nm. The metabolite peak areas were calculated relative to the peak area of the internal standard (phenacetin).

### 2.6 Ellipticine-DNA adduct detection by ^32^P-postlabelling analysis

For DNA adduct analysis cells were seeded in 75 cm^2^ flasks. After treatment the cells were washed with PBS twice and genomic DNA was isolated by a standard phenol-chloroform extraction method. DNA adducts were measured for each DNA sample using the nuclease P1 enrichment version of the thin-layer chromatography (TLC)-^32^P-postlabelling method as described previously (Stiborova et al., 2008). After chromatography TLC plates were scanned using a Packard Instant Imager (Dowers Grove, IL, USA). DNA adduct levels were calculated as described (Phillips and Arlt, 2014). Results were expressed as DNA adducts/10^8^ nucleotides.

## 3 Results

### 3.1 Cell viability after treatment with drugs

**Fig. 2:**
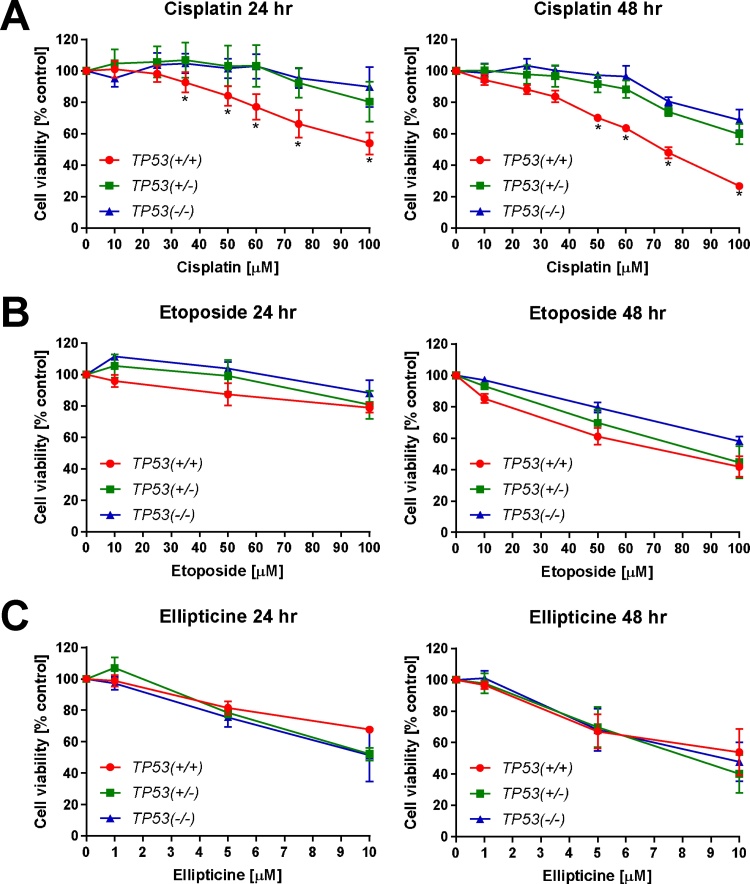
Effect of cisplatin (A), etoposide (B) and ellipticine (C) on cell viability (% control) in isogenic HCT116 cells after 24 (left panels) and 48 h (right panels) using crystal violet staining. Controls were treated with solvent vehicle only. Values are means ± SD (n = 3–6). Statistical analysis was performed by *t*-test (*p < 0.05, HCT116 *TP53(+*/*–)* and HCT116 *TP53(*–/–*)* cells different from HCT116 *TP53(+/+*) cells). (For interpretation of the references to colour in this figure legend, the reader is referred to the web version of this article.)

In initial tests the cytotoxicity of the drugs was determined in *TP53(+/+*), *TP53(+/–)* and *TP53(*–/–*)* cells after 24 and 48 h (Fig. 2).

The percentage of NaCl, used as the solvent to dissolve cisplatin varied in the culture medium. Therefore, the effect of 1.25, 2.5 and 5% of the 0.9% NaCl stock solution on cell viability was tested. NaCl had no influence on cell viability (data not shown). In contrast, exposure to cisplatin (0–100 μM) decreased cell viability; cisplatin was significantly more cytotoxic in *TP53(+/+*) cells than in *TP53(+/*–*)* and *TP53(–*/–*)* cells (Fig. 2A). After 24 h exposure to 25 μM cisplatin, *TP53(+/+*) cells showed greater sensitivity to the drug compared to *TP53(+/*–*)* and *TP53(–*/–*)* cells; cytotoxicity in *TP53(+/+*) cells was significantly different at concentrations ≥35 μM cisplatin. At 100 μM cisplatin cell viability was only 53% in *TP53(+/+*) cells, whereas in *TP53(+/–)* and *TP53(–/–)* cells viability was still 80–90%. After 48 h there was the same trend as at 24 h, with *TP53(+/+*) cells showing more sensitivity to cisplatin than *TP53(+/*–*)* and *TP53(−*/*−)* cells; cytotoxicity in *TP53(+/+*) cells was significantly different at concentrations ≥50 μM cisplatin. In *TP53(+/+*) cells viability decreased to 26% after exposure to 100 μM cisplatin whereas *TP53(+/−)* and *TP53(*−/−*)* cells showed 60–70% viability. More concentrations were chosen for testing cisplatin cytotoxicity than for the other drugs due to the non-linear decrease in cell viability with increasing cisplatin concentration and the large difference in sensitivity between the cell lines.

Treatment with etoposide for 24 h caused only a small effect on cell viability in all three cell lines (Fig. 2B); cell viability remained ∼80% at the highest concentration tested (100 μM). After 48 h, all three cell lines showed the same trend, with cell viability decreasing with increasing etoposide concentrations. *TP53(+/+*) cells appeared to be slightly more sensitive to etoposide than *TP53(+*/–*)* and *TP53(*–/–*)* cells, but not statistically significantly different. The lowest concentration of etoposide (10 μM) had little effect on cell viability, 50 μM produced 60–80% cell viability across the lines and 100 μM resulted in 40–60% cell viability.

After ellipticine exposure cell viability decreased in a dose-dependent manner, both after 24 and 48 h (Fig. 2C); treatment with 1 μM ellipticine had no effect on cell viability. It appears that at the highest concentration tested (10 μM) both *TP53(+/*–*)* and *TP53(*–/–*)* cells were more sensitive to ellipticine than *TP53(+/+* ) cells, but this difference was not statistically significant. After 24 h exposure to 5 μM ellipticine, all three cell lines showed a decrease in cell viability to ∼80% of the DMSO control, whereas at 10 μM cell viability varied between 50 and 70%. After 48 h exposure cell viability decreased to ∼70% at 5 μM ellipticine, with a further decrease to 40–50% at 10 μM ellipticine.

### 3.2 DNA damage response after treatment with drugs

**Fig. 3:**
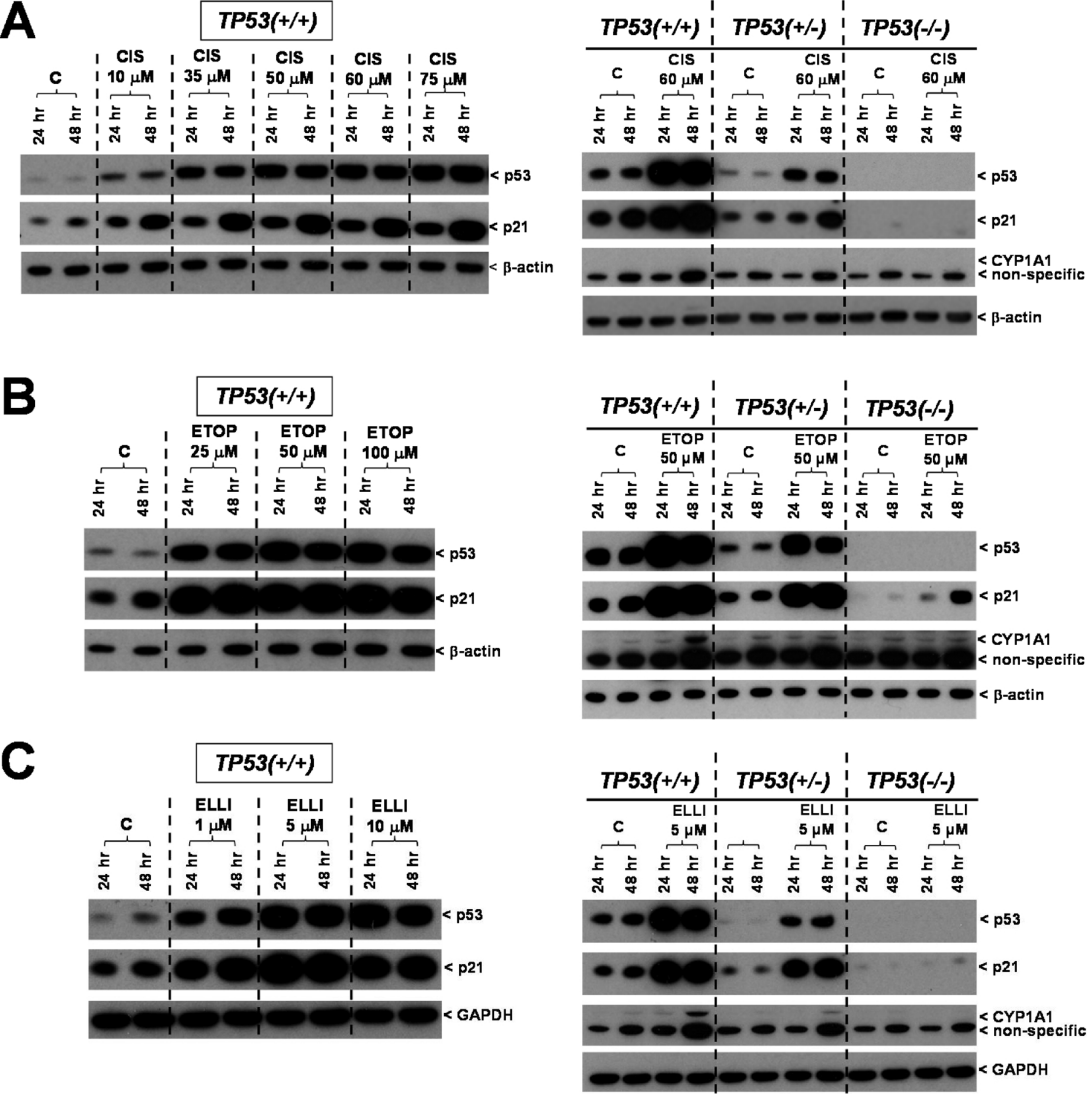
Western blot analysis of p53, p21 (CDKN1A) and CYP1A1 protein expression in isogenic HCT116 cells after exposure to cisplatin [CIS] (A), etoposide [ETOP] (B) and ellipticine [ELLI] (C) for 24 and 48 h. Based on cytotoxicity data (compare Fig. 2) and DNA damage response in HCT116 *TP53(+/+*) cells (*left panels*), protein expression in HCT116 *TP53(+/+*), *TP53(+/*–*)* and *TP53(*–/–*)* cells was compared at 60 μM cisplatin, 50 μM etoposide and 5 μM ellipticine, respectively (*right panels*). Controls (C) were treated with solvent vehicle only. Representative images of the Western blotting are shown, and at least duplicate analysis was performed from independent experiments. β-Actin or GAPDH protein expression was used as loading control.

Based on the cytotoxicity data the expression of DNA damage response proteins (p53 and p21) was assessed by Western blotting at selected concentrations of cisplatin (10, 35, 50, 60 and 75 μM), etoposide (25, 50 and 100 μM) and ellipticine (1, 5 and 10 μM) in *TP53(+/+*) cells (Fig. 3; *left panels*). The tested concentrations ranged from being non-cytotoxic to moderately cytotoxic with the aim of finding a concentration for each drug where the level of damage is high enough to induce a p53 response while most cells remain viable after 48 h.

For cisplatin, in *TP53(+/+*) cells there was a noticeable p53 induction compared to controls even at the lowest cisplatin concentration tested (10 μM) (Fig. 3A; *left panel*). At 35 μM cisplatin, p53 induction was far greater than for 10 μM, increasing further at 50, 60 and 75 μM. For all cisplatin concentrations tested, p53 levels remained constant at 24 and 48 h, whereas p21 induction was significantly higher after 48 h than after 24 h.

Taking into account the cytotoxicity data and the Western blotting results for DNA damage response in *TP53(+/+*) cells, the concentration of 60 μM cisplatin was chosen for further experiments. This is because 60 μM cisplatin strongly induced p53 and p21 and although 100 μM induced p53 to a greater extent than 60 μM (data not shown), cell viability was severely impaired at 100 μM as only ∼25% of the cells survived after 48 h (see Fig. 2A). 60 μM cisplatin produced 64% cell viability in the *TP53(+/+*) cell line, whereas *TP53(+/*–*)* and *TP53(*–/–*)* cell lines both showed 80–100% viability at that concentration. Evaluation of the DNA damage response in *TP53(+/*–*)* and *TP53(*–/–*)* cells after exposure to 60 μM cisplatin showed that as expected p53 expression was lower in *TP53(+/*–*)* cells compared to *TP53(+/+*) cells, whereas no p53 expression was detected in *TP53(*–/–*)* cells (Fig. 3A; *right panel*). As seen in *TP53(+/+*) cells, p21 expression was higher after 48 h relative to 24 h cisplatin exposure, and no p21 expression was observed in *TP53(*–/–*)* cells.

In *TP53(+/+*) cells treatment with etoposide resulted in increased p53 and p21 expression even at the lowest concentration tested (25 μM), both after 24 and 48 h (Fig. 3B; *left panel*). Expression of p53 further increased at 50 and 100 μM etoposide but no differences were observed between 24 and 48 h. The p21 expression profile was similar to that observed for p53. As 50 μM etoposide led to moderate cytotoxicity with maximal p53 expression, this concentration was chosen for further experiments. Comparison of the DNA damage response in *TP53(+/+*), *TP53(+/*–*)* and *TP53(*–/–*)* cells showed lower induction of p53 in *TP53(*+/–*)* cells relative to *TP53(+/+*) cells and no expression in *TP53(*–/–*)* cells, as expected (Fig. 3B; *right panel*). It is noteworthy that p21 induction was detectable in etoposide-treated *TP53(*–/–*)* cells, which was more prominent after 48 h compared to 24 h. This effect was not seen in *TP53(*–/–) cells treated with cisplatin (compare Fig. 3A; *right panel*), but ellipticine also showed a faint but detectable induction of p21 in *TP53(*–/–*)* cells after 48 h (Fig. 3C; *right panel*).

Expression of p53 and p21 increased in a concentration-dependent manner in *TP53(+/+*) cells after exposure to ellipticine (Fig. 3C; *left panel*). Clear induction of both proteins was visible even at non-cytotoxic concentrations (1 μM), both after 24 and 48 h. As no increase in p53 and p21 induction was seen after 10 μM relative to 5 μM ellipticine and aiming to select a concentration where 60–80% of cells remain viable, 5 μM ellipticine was used in subsequent experiments. As seen for cisplatin and etoposide, p53 expression was induced in *TP53(+*/–*)* cells after both 24 and 48 h ellipticine exposure and, as expected, p53 levels were lower to those observed in *TP53(+/+*) cells (Fig. 3C; *right panel*). In both cell lines the expression profile for p21 was similar to that seen for p53.

### 3.3 The impact of p53 function on the chemotherapeutic drug-induced expression of CYP1A1

Many PAHs including BaP are metabolised by P450 enzymes, particularly CYP1A1 (Stiborova et al., 2016; Stiborova et al., 2014b). As previous studies have shown that *TP53* status impacts on BaP-mediated CYP1A1 expression in HCT116 cells (Hockley et al., 2008; Wohak et al., 2016), we first studied the effect of cisplatin (60 μM), etoposide (50 μM) and ellipticine (5 μM) on CYP1A1 expression in *TP53(+/+*), *TP53(+/*–*)* and *TP53(*–/–*)* cells by Western blotting. Two bands were detected on the Western blot for CYP1A1; the top band is the correct molecular weight (58 kDa), and thus, the lower band is assumed to be nonspecific. This is consistent with other studies using this antibody to detect human CYP1A1 in other cultured BaP-treated human cells (Hamouchene et al., 2011; Wohak et al., 2016; Baker et al., 2018). Previous investigations in our laboratory have shown that the top band increases with higher BaP concentrations used and also that only the top band is diminished when BaP-treated cells have been transfected with *CYP1A1* siRNA (Kucab & Arlt, unpublished data).

Etoposide and ellipticine showed a clear induction of CYP1A1 expression in *TP53(+/+*) cells after 48 h but not after 24 h treatment (Fig. 3B & C; *right panels*). A weak induction of CYP1A1 protein was seen in *TP53(+/*–*)* cells after 48 h exposure to etoposide and ellipticine whereas almost no such effect was seen in *TP53(*–/–*)* cells. After exposure to cisplatin no CYP1A1 expression was observed in any of the three cell lines (Fig. 3A; *right panels*). Collectively these results indicate that etoposide and ellipticine induce expression of CYP1A1 and that this CYP1A1 induction depends on p53 function.

### 3.4 The effects of drugs on BaP-induced CYP1A1 expression and on BaP metabolism

**Fig. 4:**
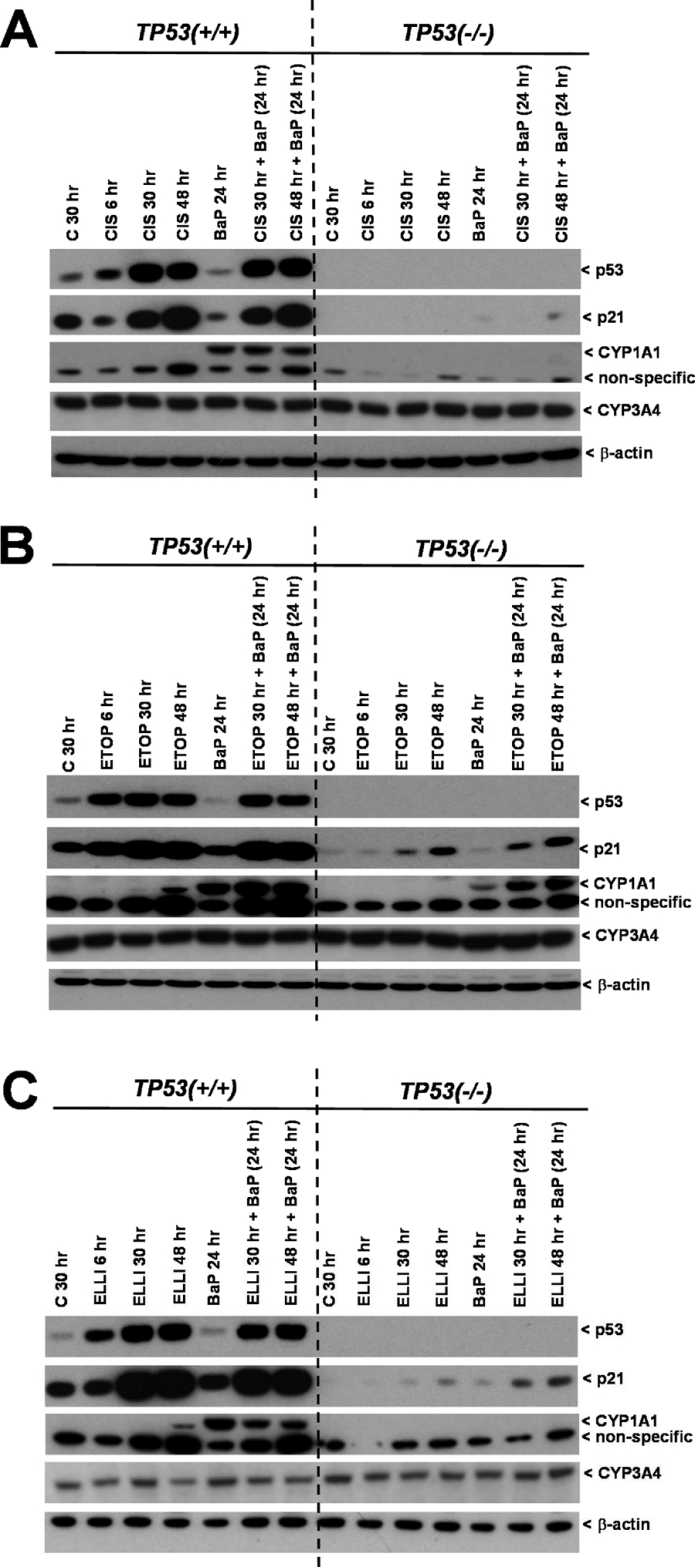
Western blot analysis of p53, p21 (CDKN1A), CYP1A1 and CYP3A4 protein expression in isogenic HCT116 cells after treatment to cisplatin [CIS] (A), etoposide [ETOP] (B) and ellipticine [ELLI] (C) and co-incubated with BaP. HCT116 *TP53(+/* *+* ) and *TP53(*–/–*)* cells were treated with 60 μM cisplatin, 50 μM etoposide and 5 μM ellipticine for 6, 30 and 48 h, respectively, or pretreated with 60 μM cisplatin, 50 μM etoposide and 5 μM ellipticine for 6 or 24 h, respectively, followed by co-incubation of the drug with 2.5 μM BaP for further 24 h. For comparison cells were treated with 2.5 μM BaP alone for 24 h. Controls (C) were treated with solvent vehicle only.

**Fig. 5:**
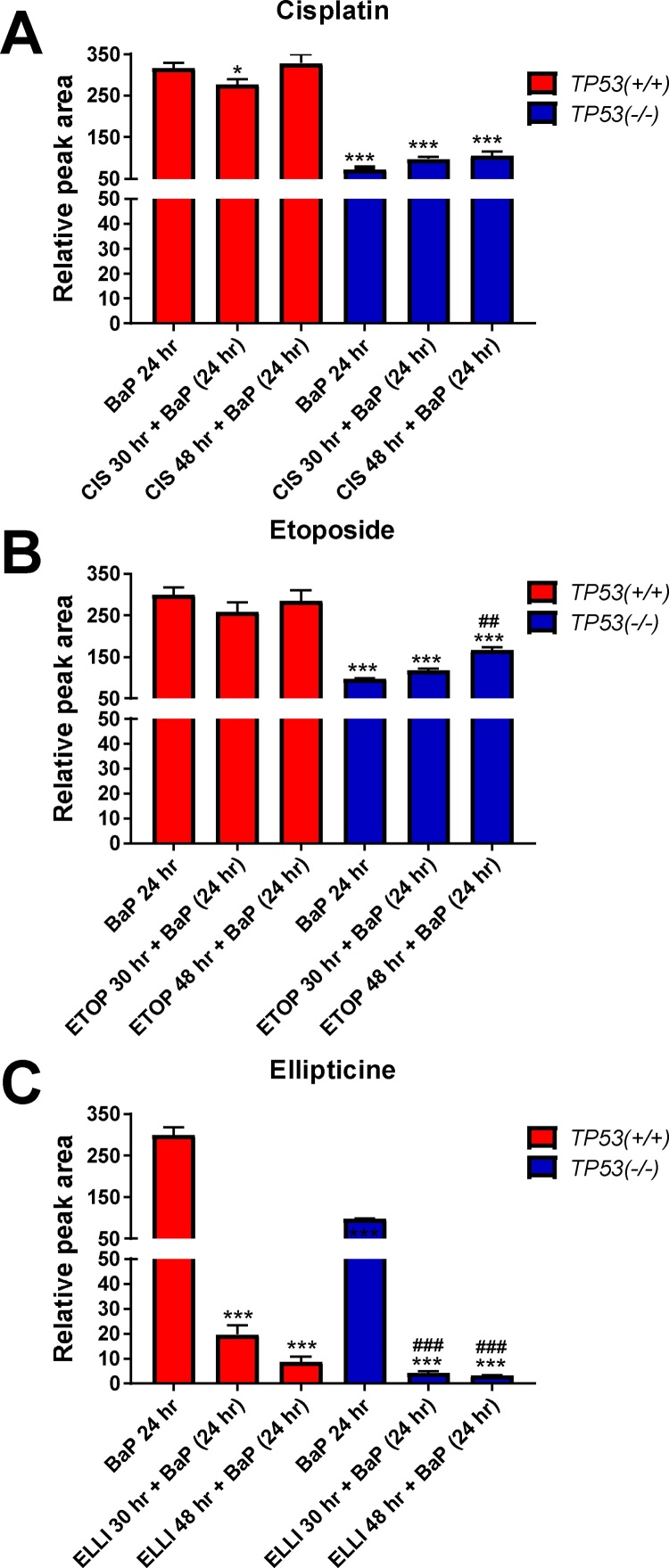
HPLC analysis of BaP-7,8-dihydrodiol in the cell culture medium of HCT116 cells after treatment to cisplatin [CIS] (A), etoposide [ETOP] (B) and ellipticine [ELLI] (C) and co-incubated with BaP. HCT116 *TP53(+/+*) and *TP53(*–/–*)* cells were pretreated with 60 μM cisplatin, 50 μM etoposide and 5 μM ellipticine for 6 and 24 h, respectively, followed by co-incubation of the drug with 2.5 μM BaP for further 24 h. For comparison cells were treated with 2.5 μM BaP alone for 24 h. Values are means ± SD (n = 3). Statistical analysis was performed by one-way-ANOVA followed by the Tukey post hoc test (*p < 0.05, ***p < 0.001, different from BaP-treated HCT116 *TP53(+/+*) cells; ^###^*p *< 0.001 different from BaP-treated HCT116 *TP53*(–/–*)* cells).

We next studied the effect of cisplatin, etoposide and ellipticine treatment on BaP-induced CYP1A1 expression (Fig. 4) and on BaP metabolism (Fig. 5) using co-incubation experiments in *TP53(+/+*) and *TP53(*–/–*)* cells. *TP53(+/+*) and *TP53(*–/–*)* cells were treated with 2.5 μM BaP in co-incubation experiments with the drugs. As before, p53 and p21 expression were determined by Western blotting (Fig. 4). Exposure to BaP for 24 h did not lead to increased p53 or p21 expression in *TP53(+/+*) cells and virtually no p21 induction was observed in *TP53(–/–)* cells. As shown before (compare Fig. 3), exposure to cisplatin, etoposide and ellipticine resulted in the induction of p53 and p21 protein levels in *TP53(+/+*) cells but co-incubation with BaP did not enhance the expression levels further (Fig. 4). In contrast, co-incubations with etoposide or ellipticine and BaP resulted in higher p21 expression in *TP53(–/–)* cells compared to each compound alone (Fig. 4B & C).

As shown previously (Wohak et al., 2016), 24 h exposure to BaP alone led to a high induction of CYP1A1 in *TP53(+/+*) cells but only low to no induction in *TP53(–/–)* cells (Fig. 4). Treatment with cisplatin did not alter BaP-induced CYP1A1 expression in *TP53(+/+*) cells (Fig. 4A). However, cells that were exposed to etoposide for 6 or 24 h and then to etoposide and BaP for another 24 h showed marked increases in CYP1A1 induction in both *TP53(+/+*) and *TP53(*–/–*)* cells compared with exposure to BaP alone (Fig. 4B**)**. Interestingly, treatment with ellipticine showed the opposite trend. In *TP53(+/+*) cells pretreated with ellipticine for 6 or 24 h and then with ellipticine and BaP for 24 h CYP1A1 expression levels decreased relative to *TP53(+/+*) cells treated with BaP alone for 24 h (Fig. 4C). These results indicate that exposure to etoposide and ellipticine can influence BaP-mediated CYP1A1 induction in a p53-dependent manner which may subsequently impact on BaP metabolism.

BaP metabolite formation was determined in the cell culture medium using HPLC analysis (Fig. 5). Again, *TP53(+/+*) and *TP53(–/–)* cells were treated with BaP in co-incubation experiments with the drugs. As a marker for BaP metabolism, the formation of BaP-7,8-dihydrodiol was measured, as studied previously (Wohak et al., 2016). This metabolite is the precursor of the reactive intermediate BPDE capable of covalently modifying DNA. BaP-7,8-dihydrodiol formation was ∼4-fold lower in *TP53(*–/–*)* cells than in *TP53(+/+*) cells (Fig. 5), confirming previous results (Wohak et al., 2016). The formation of BaP-7,8-dihydrodiol was not altered in *TP53(+/+*) cells pre-treated with cisplatin or etoposide for 24 h and then co-incubated with either drug and BaP for another 24 h (Fig. 5A & B). In *TP53(+/+*) cells treated with cisplatin for 6 h and then with cisplatin and BaP for 24 h, levels of BaP-7,8-dihydrodiol were significantly lower than *TP53(+/+*) cells treated with BaP only for 24 h (*p <* 0.05), however differences were quite small (1.2-fold). In *TP53(+/+*) cells co-incubated with ellipticine and BaP formation of BaP-7,8-dihydrodiol was substantially lower (up to ∼97% reduced) compared to *TP53(+/+*) cells treated with BaP only (Fig. 5C). Whereas pretreatment of *TP53(*–/–*)* cells with cisplatin had no effect on BaP metabolism (Fig. 5A), the formation of BaP-7,8-dihydrodiol was 1.7-fold (*p <* 0.01) higher in *TP53(*–/–*)* cells treated with etoposide for 24 h and then with etoposide and BaP for another 24 h than in *TP53(*–/–*)* cells treated with BaP only for 24 h (Fig. 5B). Similarly to the observation made in *TP53(+/+*) cells, co-treatment of *TP53(*–/–*)* cells with ellipticine had a substantial impact on BaP metabolism resulting in lower BaP metabolite levels (Fig. 5C).

### 3.5 The effect of BaP on drug metabolism

**Fig. 6:**
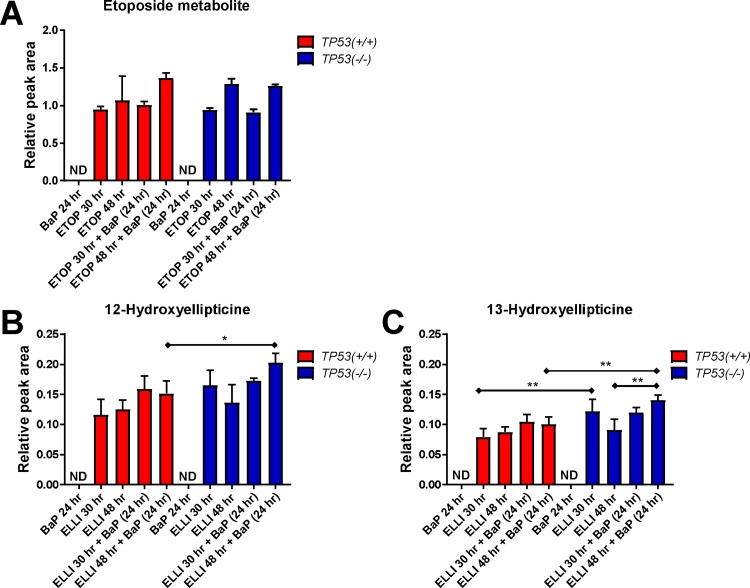
(A) Assessment of etoposide metabolism by HPLC analysis in the cell culture medium of HCT116 cells after treatment to etoposide [ETOP] and co-incubated with BaP. HCT116 *TP53(+/+*) and *TP53(*–/–*)* cells were treated with 50 μM etoposide for 30 and 48 h, respectively, or pretreated with 50 μM etoposide for 6 and 24 h, respectively, followed by co-incubation of etoposide with 2.5 μM BaP for further 24 h. HPLC analysis of 12-hydroxyellipticine (B) and 13-hydroxyellipticine (C) in the cell culture medium of HCT116 cells after treatment to ellipticine [ELLI] and co-incubated with BaP. HCT116 *TP53(+/+*) and *TP53(–/–)* cells were treated with 5 μM ellipticine for 30 and 48 h, respectively, or pretreated with 5 μM ellipticine for 6 or 24 h, respectively, followed by co-incubation ellipticine with 2.5 μM BaP for further 24 h. For comparison cells were treated with 2.5 μM BaP alone for 24 h. All values are means ± SD (n = 3). Statistical analysis was performed by one-way-ANOVA followed by the Tukey post hoc test (*p < 0.05, **p < 0.01). ND, not detected.

We further studied the effect of BaP treatment on etoposide or ellipticine metabolism by HPLC analysis (Fig. 6). For etoposide one metabolite was detectable which is probably the etoposide catechol (Zhuo et al., 2004); however further structural identification was not attempted in the present study. No significant differences in etoposide metabolite formation were observed between *TP53(+/+*) and *TP53(*–/–*)* cells under any of the experimental conditions (Fig. 6A), indicating that neither *TP53* status nor BaP co-incubation has an influence on etoposide metabolism. In order to investigate the metabolism of ellipticine we measured the formation of 12- and 13-hydroxyellipticine. Cellular responses on ellipticine metabolism were complex. Exposure of *TP53(*–/–*)* cells to ellipticine for 24 h and co-incubation of the drug with BaP for further 24 h resulted in a 1.3-fold increase (*p <* 0.05) in 12-hydroxyellipticine relative to *TP53(+/+*) cells (Fig. 6B). However, in the absence of BaP *TP53* status had no influence on the generation of 12-hydroxyellipticine. In contrast, formation of 13-hydroxyellipticine was 1.5-fold higher (*p <* 0.01) in *TP53(*–/–*)* cells compared to *TP53(+/+*) cells (Fig. 6**C**). Exposure of *TP53(*–/–*)* cells to ellipticine for 48 h and co-incubation with BaP for 24 h resulted in a 1.4-fold increase (*p <* 0.01) in the generation of 13-hydroxyellipticine relative to *TP53(+/+*) cells (Fig. 6C). There was also a 1.5-fold increase (*p <* 0.01) of 13-hydroxyellipticine in *TP53(*–/–*)* cells pretreated with ellipticine for 24 h and co-incubated with BaP for another 24 h compared to *TP53(*–/–*)* cells treated with ellipticine alone for 48 h (Fig. 6C). Collectively, these results indicate that BaP exposure led to small, but significant, alterations in the formation of 12- and 13-hydroxyellipticine in a *TP53*-dependent manner.

### 3.6 The impact of p53 function on ellipticine-DNA adduct formation and on ellipticine metabolism

**Fig. 7:**
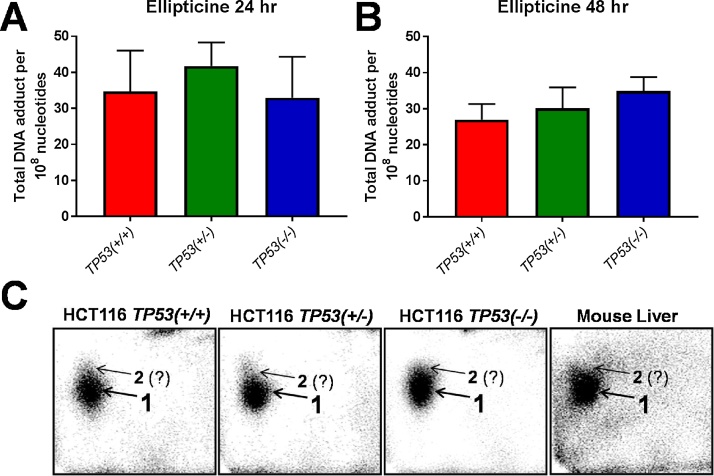
DNA adduct levels detected by ^32^P-postlabelling in isogenic HCT116 cells after exposure to 5 μM ellipticine for 24 (A) and 48 h (B). Values are the means ± SD (n = 4). Statistical analysis was performed by one-way ANOVA followed by the Tukey post-hoc test; no significant differences were observed. (C) Autoradiographic profiles of DNA adducts formed in HCT116 cells after exposure to ellipticine; the origins, at the bottom left-hand corners, were cut off before exposure. Livers of mice (on C57BL/6 background) treated with 10 mg/kg body weight for 24 h by intraperitoneal injection was used for comparison.

As the bioactivation of ellipticine can be catalysed by CYP enzymes including CYP1A1 (Kotrbova et al., 2011; Stiborova et al., 2012b; Stiborova et al., 2004) and based on the results that p53 function impacts on ellipticine-induced CYP1A1 expression, ellipticine-DNA adduct formation after 24 and 48 h was determined by the ^32^P-postlabelling method (Fig. 7). After treatment with 5 μM ellipticine the adduct pattern was qualitatively similar in *TP53(+/+*), *TP53(+/*–*)* and *TP53(*–/–*)* cells and consisted of one major and one minor DNA adduct (assigned spots 1 and 2; Fig. 7C) previously detected *in vitro* and *in vivo* by this method (Stiborova et al., 2008; Stiborova et al., 2012b). No DNA adducts were detected in untreated controls (data not shown). Because both adduct spots were incompletely separated total ellipticine-DNA adduct levels were determined. Quantitative ^32^P-postlabelling analysis showed that *TP53* status had no impact on ellipticine-DNA adduct formation under these experimental conditions (Fig. 7A & B).

Previous studies have shown that generation of adduct 1 is catalysed by a variety of CYPs including CYP1A1, but predominantly by CYP3A4, and that 13-hydroxyellipticine is a precursor in the formation of this adduct (Stiborova et al., 2012b). 12-Hydroxyellipticine is a precursor for the generation of adduct 2; the formation of 12-hydroxyellipticine is catalysed by a variety of CYPs such as CYP2C and CYP3A4 but not CYP1A1 (Stiborova et al., 2012b). Using Western blot analysis we found that CYP3A4 is expressed in HCT116 cells but that CYP3A4 expression was not influenced by drug treatment or *TP53* status (see Fig. 4). We determined the formation of 12- and 13-hydroxyellipticine by HPLC analysis in *TP53(+/+*) and *TP53(*–/–*)* cells after exposure to 5 μM ellipticine for 30 and 48 h as part of the co-incubation experiments with BaP (see details below). As shown in Fig. 6B the levels of 12-hydroxyellipticine did not differ between *TP53(+/+*) and *TP53(*–/–*)* cells after either 30 or 48 h exposure to ellipticine. The levels of 13-hydroxyellipticine were 1.5-fold (*p <* 0.01) higher in *TP53(–/–)* cells than in *TP53(+/+*) cells after 30 h, but no difference was seen after 48 h (Fig. 6C). As ellipticine-induced CYP1A1 expression was higher in *TP53(+/+*) cells than in *TP53(*–/–*)* cells and as expression only occurs after 48 h treatment (compare Fig. 3C) it appears unlikely that the observed small difference in 13-hydroxyellipticine levels between *TP53(+/+*) and *TP53(*–/–*)* cells after the 30 h treatment are linked to differences in CYP1A1 expression.

## 4 Discussion

It has been shown previously that p53 function impacts on the expression of CYP1A1 in isogenic HCT116 cells (*TP53(+/+*), *TP53(+/*–*)* and *TP53(–/–)* cells) after exposure to BaP (Hockley et al., 2008; Wohak et al., 2016). Similarly, treatment of *Trp53(+/+*), *Trp53(+/*–*)* and *Trp53(*–/–*)* mice with BaP also showed that p53 impacts on the CYP1A1-mediated metabolism of BaP *in vivo* (Krais et al., 2016a), indicating a novel function for p53 in the regulation of xenobiotic metabolism (Krais et al., 2016b). However the effect of chemotherapeutic drugs, which work by activating p53, on CYP1A1 expression is largely unknown. Therefore, three chemotherapeutic drugs, etoposide, cisplatin and ellipticine, were tested in isogenic HCT116 cells with varying *TP53* status. Our investigations not only established how these drugs could affect CYP1A1 expression in a p53-dependent manner but also focused on their influence on BaP-mediated induction of CYP1A1 and on BaP metabolism (Fig. 1). This can have important clinical implications for cancer patients with *TP53* mutations in their tumours as many of these mutations diminish or abolish the function of this tumour suppressor (Freed-Pastor and Prives, 2012).

Unlike the other compounds tested, the sensitivity of *TP53(+/+*) cells to cisplatin was more than 2-fold higher than the *TP53(+/*–*)* and *TP53(*–/–*)* cells. The same trend has been reported previously in HCT116 cell lines, where *TP53(*–/–*)* cells were significantly less sensitive to apoptosis showing that p53 is required to mediate p38alpha MAPK, via the production of ROS, causing apoptosis (Bragado et al., 2007). In contrast, results obtained in ovarian cancer cell lines demonstrated that *TP53(*–/–*)* cells responded most sensitively to cisplatin (Hagopian et al., 1999; Pestell et al., 2000), while in mouse testicular teratocarcinoma cells cisplatin treatment resulted in rapid apoptosis in *Trp53(+/+*) cells but not in *Trp53(*–/–*)* cells (Zamble et al., 1998). This shows that different cancer models respond differently to cisplatin. A possible explanation for the increased sensitivity of HCT116 *TP53(+/+*) cells could be that without p53, cell cycle arrest and p53-mediated apoptosis are impaired, potentially explaining the lower levels of cytotoxicity seen in the *TP53(+*/–*)* and *TP53(*–/–*)* cells. From the investigation of protein expression p53 and p21 were greatly induced by cisplatin in *TP53(+/+*) cells, with less expression in *TP53(+/*–*)* cells and none in *TP53(*–/–*)* cells, confirming that the latter cells have a complete knock-out of p53. Cisplatin did not induce CYP1A1 expression after cisplatin exposure in any of the cell lines up to 48 h.

With etoposide, Western blot analysis confirmed that 50 μM etoposide induced p53 effectively in *TP53(+/+)* and *TP53(+/*–*)* cells whereas no p53 response was observed in *TP53(*–/–*)* cells. Etoposide, along with other chemotherapeutic drugs such as cisplatin, has previously been shown to cause an induction of its clearing enzyme CYP3A4 via activation of p53 through DNA damage (Goldstein et al., 2013). In the latter study (Goldstein et al., 2013) a p53 binding site was discovered in the *CYP3A4* promoter, inducing *CYP3A4* transcription which potentially increases clearance of etoposide itself or a co-administered drug. A similar p53 binding site has also been found in the regulatory region of the *CYP1A1* gene (Wohak et al., 2016), and thus it is possible that through the same p53 activation process, caused by DNA damage, etoposide could also induce CYP1A1 expression. Although etoposide and CYP1A1 expression had not previously been investigated, incubation of 50 μM etoposide for 48 h resulted in CYP1A1 induction in *TP53(+/+*) cells but not in the other cell lines. Although these findings are in contrast to those observed for cisplatin they support the hypothesis that chemotherapeutic drugs like etoposide could induce CYP1A1 in a p53-dependent manner as CYP1A1 induction is absent in both *TP53(+/*–*)* and *TP53(–/–)* cells. On the other hand as results for cisplatin and etoposide diverge they also suggest that activation of p53 alone by chemotherapeutic drugs may not be sufficient to induce CYP1A1.

With the third drug ellipticine CYP1A1 expression was clearly induced after 48 h exposure in *TP53(+/+*) cells, as with etoposide, and this expression was not present in *TP53(*–/–*)* cells again implying a p53-dependent pathway.

Due to the involvement of CYP1A1 in BaP activation and previous findings demonstrating that *TP53(+/+*) cells showed a greater BaP bioactivation than *TP53(*–/–*)* cells (Wohak et al., 2016), we hypothesised that the induction of CYP1A1 by etoposide could lead to increased BaP bioactivation. To test this, the levels of CYP1A1 expression after treatment with etoposide and BaP alone and together were compared. These experiments showed that in *TP53(+/+*) cells etoposide co-incubated with BaP lead to a stronger CYP1A1 induction than in incubations with BaP alone and the degree of induction was greater in *TP53(+/+*) cells than in *TP53(*–/–*)* cells. This further supports the idea that etoposide can induce CYP1A1 via p53 activation, thereby potentially increasing BaP bioactivation. In order to test whether this increase in CYP1A1 expression in the co-incubation experiments actually resulted in an increase in BaP bioactivation, levels of BaP-7,8-dihydrodiol, a precursor of the DNA-reactive intermediate BPDE, were measured. In *TP53(+/+*) cells no increase in the formation of this BaP metabolite was found, suggesting that the increase in CYP1A1 expression was too small to see a difference. In contrast, in *TP53(*–/–*)* cells the extent of BaP-7,8-dihydrodiol formation was greater in the co-incubation experiments which was in line with a higher induction of CYP1A1 under these conditions. As there is also an increase in CYP1A1 expression in the co-incubation experiments in the *TP53(*–/–*)* cell line, there must also be a p53-independent pathway that etoposide is influencing which would require further investigation.

Ellipticine, like BaP, undergoes metabolic activation by CYPs such as CYP3A4 or CYP1A1 in the presence of cytochrome *b_5_*, in order to bind to DNA (Kotrbova et al., 2011; Stiborova et al., 2012a; Stiborova et al., 2004). However, in contrast to results seen for BaP in HCT116 cells (Wohak et al., 2016), *TP53* status had no impact on ellipticine-DNA adduct formation in these cells, which supports previous findings (Stiborova et al., 2012b) that CYP3A4 is more prominent than CYP1A1 in catalysing the bioactivation of ellipticine. As indicated above a previous study (Goldstein et al., 2013) showed that CYP3A4 expression is induced by chemotherapeutic agents such as cisplatin and etoposide by activating p53 and it could be hypothesised that ellipticine may behave similarly. Using Western blotting analysis we did not find an impact of cellular *TP53* status on CYP3A4 protein levels in HCT116 cells after exposure to cisplatin, etoposide or ellipticine. The similar expression levels of CYP3A4 in *TP53(+/+*) and *TP53(–/–)* cells are also in accordance with the observed similar ellipticine-DNA adduct levels in both cell lines.

Previous studies in rats have shown that ellipticine and BaP both induce CYP1A1 expression thereby increasing their own bioactivation (Aimova et al., 2008). However, studies in human cells to determine whether BaP and ellipticine influence CYP1A1 induction when both are incubated together have not previously been reported. As ellipticine alone induces CYP1A1 in this cell model, as does BaP, the assumption might have been that co-incubation of ellipticine together with BaP results in even higher CYP1A1 expression thereby increasing BaP bioactivation. In contrast, our results show a decrease in CYP1A1-mediated BaP oxidation activity when BaP and ellipticine are present in the cell, thus metabolic bioactivation of BaP was reduced in these cells. Ellipticine seems to be a better substrate for induced CYP1A1 which competes with BaP to its binding to the active centre of CYP1A1, thereby decreasing the formation of BaP-7,8-dihydrodiol.

The overall aim of this project was to investigate the effect that chemotherapeutic drugs have on the CYP1A1-mediated metabolic activation of BaP. As BaP is found in tobacco smoke (Alexandrov et al., 2016; Kucab et al., 2015; Nik-Zainal et al., 2015), any relationship found between the chemotherapeutic drugs and BaP activation could have health implications for tobacco smokers receiving treatment for cancer. This may be important for the progression of the primary tumour formed including the potential formation of metastases (i.e. formation of secondary tumours) and the efficiency of treatment including the possible reoccurrence of tumours after treatment. It is also noteworthy that many patients are still smokers when they suffer from cancer. In a previous study (Petros et al., 2012), the effect of tobacco smoke on the metabolism of chemotherapeutic drugs was investigated, showing that it induced CYP1A2, which in turn increased the metabolism of the kinase inhibitor erlotinib leading to a 24% faster clearance of the drug in smokers compared with former or never smokers and reducing its efficacy. It has also been shown that cigarette tar can increase CYP3A4 activity, the main enzyme responsible for the metabolism of many chemotherapeutic drugs, further linking smoking to an increased clearance of chemotherapeutic drugs (Kumagai et al., 2012). Our study provides additional evidence that the etoposide and ellipticine impact on CYP1A1-mediated BaP metabolism, whereas cisplatin shows no impact. Actually our results seem to indicate that ellipticine treatment would offer protection against BaP-induced DNA damage for smokers during chemotherapy; however, further studies will need to clarify the potential impact of BaP on ellipticine metabolism *in vivo* and whether it affects the efficacy of chemotherapy.

## 5 Conclusion

We found that both etoposide and ellipticine had an effect on CYP1A1 expression whereas cisplatin did not. This suggests that etoposide and ellipticine may share a common pathway on influencing CYP1A1 expression via p53 activation that differs from that of cisplatin, possibly due to their shared role as topoisomerase II inhibitors. However, whilst etoposide and ellipticine both induced CYP1A1 expression, the co-incubation experiments with BaP produced opposing results; therefore the underlying mechanism of how both drugs regulate BaP-mediated CYP1A1 expression must be different. As both drugs influence CYP1A1 in BaP co-incubations differently, it showed that the interaction between the drugs and CYP1A1 is more complex than first thought, and it is not simply an induction of CYP1A1 via p53 activation alone. Another explanation might be that in addition to activating transcription, p53 can also repress target gene expression. Our results could be relevant for smokers, who are continuously exposed to increased levels of BaP via tobacco smoke, with different treatments potentially influencing their susceptibility to BaP-induced DNA damage. Whereas treatment with cisplatin and etoposide had virtually no influence on CYP-catalysed BaP metabolism, ellipticine treatment had a strong impact. Our study provides evidence that more consideration should be given to potential drug-environment interaction during chemotherapy. In addition, this study and previous findings in our laboratory show that CYP1A1-mediated bioactivation of BaP depends on p53 function highlighting the need to consider gene-environment interactions.
